# Recognizing Zooeyia to Promote Companion Animal Welfare in Urban Bangladesh

**DOI:** 10.3390/ani13091523

**Published:** 2023-05-01

**Authors:** Abu-Hena Mostofa Kamal, Colleen Anne Dell, Timothy Kang

**Affiliations:** 1 Department of Sociology, University of Saskatchewan, Saskatoon, SK S7N 5A2, Canada; colleen.dell@usask.ca (C.A.D.); tim.kang@usask.ca (T.K.); 2 Department of Humanities, Khulna University of Engineering and Technology (KUET), Khulna 9203, Bangladesh

**Keywords:** companion animal, animal welfare, Bangladesh, zooeyia, stigma

## Abstract

**Simple Summary:**

The practice of companion animal keeping is growing in urban Bangladesh. People are beginning to identify how the addition of a companion animal in their lives can benefit their health. There are also longstanding concerns about animal welfare in Bangladesh. Interviews with companion animal owners, companion animal sellers and veterinarians in two major urban Bangladesh cities offer insights into the benefits and challenges of companion animal ownership. We suggest four ways to apply this understanding to promote companion animal welfare in Bangladesh.

**Abstract:**

The One Health concept of zooeyia refers to the benefits of companion animals in human health and is gaining global research attention. This exploratory study aimed to understand contemporary experiences and perceptions of the social benefits and challenges of living with a companion animal in urban Bangladesh. Thirty-five qualitative interviews were conducted with companion animal owners (20), animal sellers (10), and livestock service department officers (5) from two major cities in Bangladesh, Dhaka and Khulna. Thematic analysis found that historically, animals had a utilitarian purpose, such as livestock for food and dogs for security. The role and perceptions of companion animals began to change for some around the turn of the century. Today, companion animal caretakers report social, psychological and physical health benefits from integrating companion animals into their lives. They also report that companion animal ownership can contribute to social problems due to the prevailing stigma against companion animals. This is rooted in the continued utilitarian role attached to companion animals by the majority of the Bangladesh population as well as religious-based non-acceptance. As a result, the *Animal Welfare Act* (2019) is not well implemented, posing a key concern for companion animal welfare. To tackle this, we propose various ways in which the emerging concept of zooeyia can help promote the welfare of companion animals by challenging the stigma associated with them in Bangladesh.

## 1. Introduction

The benefits of companion animal ownership on human health is gaining global attention [1,2]. We use the term ownership in this paper because it indicates the possession of companion animals by humans, but at the same time it is important to recognize this is problematic for several reasons and may be a contributor to companion animal welfare concerns. In 2011, the concept of zooeyia was introduced to the One Health Framework; One Health recognizes the interconnection between human, animal, and environment health [3]. Zooeyia refers to the “positive benefits to human health from interacting with animals, focusing on the companion animal” [4]. Awareness of zooeyia was amplified during the COVID-19 pandemic, where forced isolation of many people increased the time they spent with a companion animal in their homes [5,6]. 

Research indicates that over half of North American households, where the majority of research is concentrated, have at least one companion animal and most owners identify them as a family member [4,7,8]. This is a growing trend within developed countries [9,10,11]. Hodgson and Darling (2011) identify four key ways companion animals can benefit human health: as builders of social capital, agents of harm reduction, facilitators of behaviour change and as a part of treatment plans [12]. For example, an emerging body of study suggests companion animals can help increase physical activity among aging individuals [13,14] and improve the immune function of children in a household [15]. Research has also identified the specific role of companion animals in reducing human loneliness [1,16,17].

With the rapid increase in companion animal ownership across the globe, there is an increased need to focus on companion animal welfare, especially given that cruelty toward companion animals is a serious global concern [18]. Research shows that thousands of companion animals are abused each day, although most abuse is undetected because it occurs in private spaces [19]. Dogs, cats, and horses tend to be the most common documented victims of violence [20]. That said, the types of abuse against companion animals and the reasoning behind them varies across demographics and countries. In the United States, for example, negligence and abandonment have been identified as common types of violence against companion animals, with the primary reason being lack of awareness and knowledge about companion animal maintenance [20]. For instance, a 2018 statistic indicates that approximately 32% of the deaths of companion animals in the United States were due to negligence [19]. Negative stereotyping and myths about companion animals (e.g., black cats are bad luck, bully breed dogs are aggressive) and the resulting stigma can contribute to a lack of education and resources to safeguard for proper animal care and treatment [21]. 

Asian countries have a storied history of companion animal abuse, with much of it unreported [22]. Studies show that poverty and a lack of animal cruelty laws in the majority of Asian countries are the key reasons behind animal abuse [22]. Specific to Bangladesh, there is no exact statistic on the companion animal population in the country, but it appears to have increased in the past few decades [23]. The Bangladesh *Business Post* newspaper indicates that live pet animal and bird importation increased in 2020–2021, as has spending on companion animals and the number of pet shops across the country [24].

There is also no accurate statistic associated with companion animal abuse or welfare in Bangladesh [25]. This is the case even though the 1920 Bangladesh *Cruelty to Animals Act* was revised and updated to the *Animal Welfare Act* in 2019. The new *Act* aims to “ensure the proper treatment and responsible rearing of animals and of preventing cruel treatment” [26]. Newspaper articles indicate a lack of awareness about animal welfare among companion animal owners [25,27,28]. Animal welfare groups such as Obhoyaronno-Bangladesh Animal Welfare Foundation and Guardians of Paws and Claws have documented companion animal abuse [29,30]. Animal shelters offer additional insights on the concerning state of animal welfare in Bangladesh, including a general lack of empathy among community members for companion animals [31]. Harmful stereotypes and the resulting stigma can contribute to a lack of legal protections and/or implementation for companion animals [32]. Educating the wider population about animal welfare is a major challenge as well as a dire need. 

To our knowledge, this exploratory study is the first of its kind to attempt to gain insight on contemporary companion animal welfare in Bangladesh by investigating contemporary experiences and perceptions of the social benefits and challenges of owning companion animals in two urban centres, namely Dhaka and Khulna. Based on this insight, we offer several suggestions for how companion animal welfare may be promoted through the emerging recognition of zooeyia in Bangladesh, that is, the positive impact of companion animals in human health. 

## 2. Materials and Methods

### 2.1. Study Areas

We purposively selected Dhaka and Khulna—both metropolitan areas of Bangladesh—for this study given that the international literature suggests there is a high prevalence of loneliness and fragile social interactions among people living in densely populated cities, which may result in a greater desire for animal companionship [33,34,35]. Dhaka is the capital of Bangladesh and the most densely populated city in the country. It is also the 6th most populous city in the world, with approximately 23,234 people living per square kilometre [36,37]. Khulna is the 3rd largest and fastest-growing city in Bangladesh [37].

### 2.2. Data Collection

We applied a combination of snowball and purposive sampling techniques to recruit a total of 35 participants for the study. Our main recruitment strategy was to select participants based on referral from other participants [38]. To begin, the first author on this paper (AHMK) identified potential participants for the study from his own network. These participants then referred others who might be interested. We then applied a purposive sampling technique to include participants with diverse socio-demographic characteristics such as age, gender, religion, education, marital status and economic status. We also shared our recruitment poster on the first author’s Facebook site. The inclusion criteria for companion animal owners were: having an animal in their care for at least one year, living in Dhaka or Khulna during the data collection period, being a Bangladeshi citizen, and being at least 18 years of age. Animal sellers included either owners or employees of a pet animal store and professional companion animal breeding farm owners. All animal seller participants had at least one year of experience selling companion animals. Livestock service department officers had to have been in their employment positions for at least one year. Informal observations at animal stores, farms and households were also made by the interviewer, including non-verbal cues and/or gestures of the participants. These were recorded by hand in the interview notes, both during and immediately after the interview. These observations helped to document attachment with and attitudes of the participants toward companion animals, specifically among the companion animal owners. It was also an effective strategy to document the conditions of companion animal seller and breeder spaces, including stocking density, cleanliness and types of care towards companion animals.

Data were collected between 20 December 2021 and 2 February 2022 through face-to-face in-depth interviews with companion animal owners (20) and key informants, including animal sellers (storekeepers and breeders) (10) and livestock service department officers (veterinary surgeons, 5). The interviews were conducted by the primary author on this paper (AHMK), a resident of Bangladesh with no prior companion animal ownership himself.

A semi-structured interview guide with open-ended questions was developed for each of the three target participant populations of the study. Given the lack of published information on companion animal ownership and welfare in Bangladesh, we formulated the interview guide based on related literature and studies from across the globe [39,40,41]. The guide’s main focus is the contemporary role of companion animals in urban Bangladesh and the perceived benefits and challenges of human–companion animal interaction (see Supplementary Materials). The three population-specific interview guides were pre-tested with companion animal owners (3), animal sellers (2) and a livestock service department officer. 

### 2.3. Data Management and Analysis

All the interviews were conducted in Bangladeshi and audio-recorded by the first author (AHMK). The average lengths of the interviews were 54.25 min (ranging from 20.51 min to 124 min) with companion animal owners, 29.40 min (ranging from 15 min to 61 min) with sellers and 25.54 min (ranging from 18.48 min to 31.44 min) with livestock service department officers. The interviews were transcribed verbatim in Bengali and then the interview transcriptions were translated into English. We used NVivo (version 12) as the primary organizational software. Data analysis was guided by Braun and Clarke’s (2006) six-phase framework for thematic analysis, including familiarization, coding, generating themes, reviewing themes, defining and naming themes and writing up. The primary author (A.-H.M.K.) undertook the data analysis. An open coding approach was applied to analyse the data, with no prior code list identified. Rather, the codes were developed based on the research focus and as we worked through the coding process. The second and third authors (C.A.D. and T.K.) were consulted throughout the coding process for feedback and insights. Additional codes were included based on field notes and unstructured observation. Each interview was analysed separately and the conclusions were drawn collectively based on the perceived benefits and drawbacks of companion animal ownership on human health. 

### 2.4. Ethical Considerations

The research protocol [Beh#2973] was reviewed and approved by the Behavioural Research Ethics Board at the University of Saskatchewan. The consent form was sent to each participant by email at least one day before the interview. The first author also explained the objectives and ethical considerations of the study to each of the participants in-person immediately prior to conducting the interview, and informed written consent was collected. It is important to note that the interviews were conducted during the COVID-19 pandemic. The interviewer supplied facemasks and hand sanitizers to all participants. The use of a facemask, hand sanitation at frequent intervals and the maintenance of social distance (2 m apart) was mandatory in each interview scenario. 

## 3. Results and Discussion

### 3.1. Participant Characteristics

Of the 20 companion animal owners, 11 were male, 10 were between 18 and 30 years of age, 9 were married, majority (15) had attained a Bachelor of Arts or higher degree, 11 were employed and 15 were Muslim. Of the 20 owners, 12 reported owning their companion animals between 1 and 3 years and 13 reported renting their houses. The average monthly income of the participants was USD 1369.81 (see Table 1). As a note, the average per capita monthly income in Bangladesh is USD 226.52 [36,37].

Among the animal sellers (10), six represented animal breeding farm owners (i.e., wholesale) and four were employees of an animal store (i.e., retail). All of the sellers were male and seven were Muslim. Two animal sellers had completed a Bachelor of Arts or higher level of education. Out of the six animal breeding farms, three bred dogs and the remaining bred cats. The storekeepers reported selling various types of animals, including dogs, cats, rabbits and aquarium fish. All the livestock service department officers were male veterinary surgeons. The average duration of time they had worked in the sector was 21 years, with a minimum of 8 and a maximum of 33 years (see Table 2).

### 3.2. Changing Role and Perception of Companion Animals in Bangladesh

#### 3.2.1. The Past and Its Influence on the Present

Our participants contextualized their experiences with and perceptions of the contemporary role of companion animals in Bangladesh by sharing their thoughts about the historical role of animals more generally, as well as companion animals. They collectively shared that prior to around 2000, the majority of Bangladesh households owned livestock, including cattle, buffalos, goats, chicken, ducks and hens as working animals in agricultural fields (e.g., to tend the land) as well as to meet the nutritional demands of family members (e.g., milk, egg and meat). Some participants shared that women, in particular, owned ducks, chickens and goats as a source of household income. This finding echoes the existing literature on the historic role of animals in Bangladesh, which outlines their utilitarian purpose [25,42,43,44,45].

All of the companion animal owners in our study reported that in the past, almost every rural household in Bangladesh had one or more stray dogs or cats associated with it. They shared that people fed the dogs and cats so they would stay around to protect the households from intruders and rodents. Like livestock, these animals had a utilitarian purpose. All companion animal owners shared that this utilitarian role continues today [46]. A 58-year-old veterinarian said, “[many] people, especially the non-companion animal owners, argue that animals cannot be companion to humans. For them, animals, like dogs can be utilized to guard households. Likewise, cats can help to safeguard grains and other valuable stuffs at home by killing rats and other insects.” He said that the Bangladesh media had recognized a fairly recent transition in the role of stray community dogs in particular, with them being identified by some as companion animals [47].

Our participants shared that many people owned caged wild birds as companion animals in the past in both rural and urban Bangladesh. The owning of birds was due to space limitations in dwellings as well as the influence of religious beliefs. All the companion animal owners, sellers and livestock service department officers mentioned that people did not own dogs because they were haram (forbidden) in Islam. A 36-year-old female companion animal owner stated:

“…historically individual houses are very rare in our urban settings. It was one of the reasons why people living in apartments did not own companion animals. Also, there are some socio-cultural barriers, such as religious beliefs, that prohibited people from owning non-human animals in living rooms.”

Religious forbidding of the ownership of companion animals, especially dogs, continues to the present day in Bangladesh [10,25,48]. Similar to other Muslim-majority countries, the people of Bangladesh, and especially older adults, do not allow dog ownership or dogs inside a dwelling, except for security purposes [49,50,51,52]. There is growing debate in the literature among pro-dog and anti-dog Islamic scholars. In contrast to the anti-dog scholars, drawing on the Hadith, the pro-dog scholars argue that Islamic sentiments are being incorrectly applied against dogs [53,54,55]. The anti-dog scholars capitalize on the religious beliefs and sentiments of the majority of people and claim that owning a dog as a companion animal has been prohibited in Islam.

#### 3.2.2. Current Day

Participants in our study shared that since the turn of the century, the companion-animal role has evolved in Bangladesh, with some recognition of their potential positive benefits to human social, psychological and physical health. This aligns with research findings in North America and other parts of the world [56,57]. Still, almost all of our study participants mentioned that the vast majority of people in Bangladesh are of the position that it is wasteful to invest money in non-human animals while humans suffer in the country.

Reflecting on their experiences with and perceptions of companion animals, the majority of companion animal owners in our study shared that interaction with their companion animals benefits their psychological health. They specifically shared that it reduces their sense of loneliness and controls stress, as well as depression, in their lives. A 38-year-old cat owner shared:

“I am a service holder. Most often I have extreme workload at office. Further, I stay alone at my residence given that my wife lives in another town. So, interaction with my cats help me to de-stress myself as well as gives a feelings like someone is around me.”

Some participants also reported that owning a companion animal benefits their physical health by requiring them to be active because of the associated caretaking duties. A 62-year-old companion animal owner shared: “I am retired person. I have also diabetes. My dog helps me to go for walk everyday in the morning and evening. I also wash them, clean her potty, and take her to veterinarian hospital for regular check-up and vaccination. All these helps me to be physically active.” A few participants shared that they quit smoking in consideration of the harmful second-hand impacts on their companion animals.

All of the participants in the study reported that owning companion animals is beneficial to their social networks and interactions, both online and in person. For example, they shared that there are several “animal lovers’ pages” on social media platforms, specifically Facebook and WhatsApp. Members of these online groups organize animal shows and meet and greet events. Some share information about cases of violence against animals, including the killing of stray dogs and cats by city corporations and individuals, and the violation of the animal welfare law in Bangladesh. A 36-year-old companion animal owner commented:

“We the companion animal owners organized several processions against mass culling of stray dogs in our town. Companion animal owners and activists from other cities also participated in those movements. Finally, we did a press conference where we recommended updating the hundred years old animal welfare act in Bangladesh. Our movements were successful and we believe the *Animal Welfare Act 2019* is one of the successes of our movements.”

Participants identified the evolving role of companion animals in Bangladesh as being particularly evident during the COVID-19 pandemic. Most veterinary surgeons and some companion animal owners observed that the number of people owning companion animals increased at the start of the pandemic to combat isolation. A 45-year-old female dog owner shared that she, along with her family members, purchased a dog during the pandemic because social interactions were restricted. She reported that her companion dog helped reduce her loneliness and mental depression. A 35-year-old veterinary surgeon shared:

“I have seen YouTube videos where psychiatrists are saying companion animals can help to reduce loneliness. They also suggest that animals’ companionship can help children to get rid of from substance use, addiction to social media, smartphone and computers. These are the reasons, I think, why parents encouraged their children to own companion animals during the pandemic.”

While studies across the globe have identified a positive impact of human–companion animal interactions on the mental wellbeing of owners during the COVID-19 pandemic [58,59], others have found limited or no impact [60,61]. Although larger-scale studies have yet to be conducted in Bangladesh, our exploratory results suggest there may be important, albeit qualified, perceived positive effects of companion animal ownership in the Bangladeshi context.

Our study participants shared that Western culture, specifically through social media channels, has influenced the changing role of companion animals in Bangladesh. In fact, all of the veterinary surgeons in our study shared that traditional views toward companion animals are gradually changing due to the availability of information about human–companion animal interactions in Western countries. Research originating in Scotland notes that individuals’ perceptions of animals can be influenced by social media, with other researchers identifying that the emotional reactions animal elicit on social media is a facilitator of this [62,63,64]. Some companion animal owners reported that they felt their goodness as people was recognized for owning companion animals. A 61-year-old cat owner said, “…people admire seeing my photo with my cats on Facebook. One day a person said, you must be so kind since you are kind towards animals.” One of the companion animal owners extended this to suggest that owning an animal helped to create a social media identity for them, for example, as an “animal lover”.

### 3.3. Challenges of Owning Companion Animals in Urban Bangladesh

The most common companion animal ownership challenge mentioned by the majority of our study participants is the harmful impact of stigma. This is foremost related to zoonotic disease transmission and living with companion animals.

Our study participants shared that there is fear of zoonotic disease transmission (from animals to humans) in Bangladesh. Two dog owners commented that people consider animals and animal owners to be “dirty” and “unhygienic.” All but one participant mentioned a concern about the risk of disease transmission from humans to animals, and in fact, one companion animal owner and one seller erroneously shared that disease cannot be transmitted from humans to animals. All the veterinary surgeons shared that the possibility of zoonotic disease transmission is a valid concern in Bangladesh given the lack of consistent and preventative hygienic practices among companion animal owners. One veterinary surgeon claimed that only about one-third of companion animal owners in Bangladesh are knowledgeable about zoonotic disease transmission and vaccinate their companion animals. A 58-year-old veterinary surgeon said, “making both animal owners and non-owners [aware] is important, in order to limit the transmission rate of zoonotic diseases as well as to control the fears about animals”. There is a need for companion animals in Bangladesh to be vaccinated to counteract zoonotic-related fears and the consequent poor treatment and stereotypes of companion animals [65].

All the companion animal owners mentioned conflicts with landlords and neighbours because of their companion animal ownership. They reported that neighbours were concerned about noise and the cleanliness of their dwellings. A 23-year-old male dog owner mentioned that one of his neighbours poisoned and killed his dog because of his dog’s barking at night. A 35-year-old female dog owner said that she ignores what others think about her and does not react because it could impact her relationship with her neighbours. Further, a 26-year-old female dog owner said, “in my town, it is almost impossible to find an apartment to stay in with companion animals. It seems like having a companion animal is like a sin.” Indeed, companion animal owners’ difficulty with renting because of the stigma attached to companion animal ownership is true in many parts of the world [66,67].

Many companion animal owners mentioned that the negative stigma attached to companion animal ownership limited their social interactions. They shared that some close relatives and friends do not visit their dwellings because an animal lives in it. Others shared that people will not eat food at their home because of the possibility of contamination with their companion animal’s fur. A 35-year-old animal companion owner of a cat stated:

“…my brothers do not come to my house because of owning cats. I cannot organize any social gathering at my home given that there are many people who are afraid of [my] dogs. Thus, I feel uncomfortable inviting anyone at my home or offering them food because they might deny.”

Over half of the companion animal sellers mentioned that they do not sell dogs given that people criticize “earning a livelihood by selling an animal like a dog”. Two animal sellers shared that they do not sell dogs at the local market because buying and selling dogs is prohibited by the market committee. A 49-year-old companion animal seller shared that “animals are not treated with respect. Many people hate animals claiming that they are dirty. As a result those who are involved in animal related businesses are treated as the lower class citizens.” Bangladesh is not the only country with negative views of dogs and other companion animal ownership [68,69], but the socio-historic context of Bangladesh nonetheless presents companion animal owners and sellers with significant structural challenges.

### 3.4. Companion Animal Welfare Concerns in Bangladesh

Our study participants were specifically asked about companion animal welfare concerns in Bangladesh. Informal observations were also made in the households, animal markets and farms where the interviews took place. Two primary and interrelated concerns were identified: the challenges of social–structural limitations in Bangladesh and the occurrence of animal abuse. Both can amplify the negative stigma attached to companion animals and contribute to the consequent inadequate implementation of the *Animal Welfare Act*.

Majority of the animal owners and sellers mentioned social–structural limitations as negatively impacting animal welfare in Bangladesh. They identified limited open spaces in urban areas to walk companion animals, limited number of animal shelters to house stray animals and inadequate veterinary treatment facilities alongside a lack of veterinarian expertise to treat companion animals. This is not surprising given the concerning poor treatment of farm animals in utilitarian roles [70], which would be similar to the current primary role of companion animals. In fact, most of the companion animal owners reported that veterinarians are often careless when treating sick and injured animals. They shared that veterinarians infrequently touched the animals in their care. One of the animal owners shared: “…my dog died due to the wrong treatment of a veterinarian. Since then I do not go to veterinary hospital. Rather, I feed my dogs homeopathic medicine in case they become sick.” Lack of expertise among veterinarians is a problem observed in many countries [71,72]. Consequent homeopathic treatment of animals is also evidenced in other countries [73,74]. Professional and adequate assessment and treatment is important for companion animal welfare and zoonotic disease management [70].

A key social–structural limitation influenced by stigma and identified by our study participants is that the *Animal Welfare Act* is not well implemented in Bangladesh. All the veterinary surgeons suggested that because of this people do not rely on the *Act*’s implementation. To explain, the livestock staff shared that companion animals are required to be registered at the sub-district level with the livestock service department, but the majority of owners do not. A 32-year-old veterinary surgeon said the reasons for not doing so are that there is no attached penalty and owners do not find any benefit in registering their companion animals. However, most of the veterinary surgeons emphasized the need to register companion animals because it could assist the livestock service department with keeping track of the number of companion animals, animal illnesses and could support investigating reasons for companion animal deaths. A 58-year-old veterinary surgeon shared, “[we] often hear about brutality against stray animals. Meanwhile, we barely hear anything about violence against companion animals because it remains unreported. Also, it occurs at the owners’ personal space. Therefore, our department, or animal activists, cannot interfere.”

In fact, the *Animal Welfare Act* indicates that proven violence against an animal can result in six months of imprisonment [26]. All the veterinary surgeons recommended strict implementation of the *Act.* Meanwhile, most of the companion animal owners reported the mishandling and abuse of companion animals by breeders and sellers. They shared that in order to make a profit, sellers too frequently bred companion animals, fed them poorly, and did not treat them when ill. A 35-year-old female dog owner shared, “I went to the largest animal wholesale market and found most of the dogs and cats cannot stand properly because they are frequently used for breeding purposes. I could not control my tears seeing the inhuman conditions of the animals in the market. I have never visited that market since then given that it is not possible to tolerate the inhuman conditions of the animals”. The abuse of animals was also mentioned as a result of anti-dog religious Muslim sentiment. However, as pro-dog scholars are increasingly sharing, Islam does not permit cruelty toward any living animal [75,76].

Further, informal observations during the interviews documented that the majority of companion animal owners kept their dogs and cats properly in separate locked rooms or iron cages. During the interviews with the sellers and breeders, it was observed that all the sellers, and all but one breeder, kept different types of animals (i.e., birds, cats, dogs, rabbits) in side-by-side iron cages. The animal sellers reported that the livestock services department requires them to separate the cages of different animal species. However, due to space limitations in their stores, they keep different species in side-by-side cages. During the interviews, the sellers restricted the interviewer from taking any photographs of their stores, with one study participant indicating “…it is not allowed [to take photographs and videos] by the market committee.”

## 4. Conclusions and Suggested Next Steps

This study focused on understanding companion animal welfare in Bangladesh by exploring the benefits and challenges of owning companion animals in two urban centres. We documented the evolving role of companion animals in Bangladesh based on our study participants’ experiences with and perceptions of companion animals. It appears that the number of people owning companion animals is increasing in Bangladesh, along with a recognition of zooeyia. Majority of our participants mentioned the social, psychological and physical health benefits of companion animal ownership. Our participants also identified key challenges to owning companion animals in urban Bangladesh due to the negative stigma associated with companion animal ownership. According to our participants, this is rooted in the continuing utilitarian role attached to companion animals by majority of the Bangladesh population and religion-based non-acceptance. The consequent lack of implementation of the *Animal Welfare Act* was identified as a key companion animal welfare concern.

According to the American Veterinary Medical Association, “[p]rotecting an animal’s welfare means providing for its physical and mental needs”[77]. Recognition of zooeyia in Bangladesh offers an opportunity to combat stigma and the harmful consequences associated with companion animal ownership. Quite simply, a companion animal cannot benefit human health if they are not well cared for themselves. For example, it would be contradictory to consider the positive impact of petting a dog for human benefit (e.g., to increase stress-reducing hormones), while the dog themself is not well. In fact, companion petting an unhappy dog would most likely not result in a human health benefit. According to Proctor (2012), “[d]emonstrating objectively what animals are capable of is key to achieving a positive change in attitudes and actions towards animals, and a real, sustainable difference for animal welfare”. The findings of this study helped identify four ways in which recognition of zooeyia, that is, the ability of companion animals to benefit human health, can help promote the welfare of companion animals in Bangladesh by challenging the stigma associated with them.

1. Zooeyia is a newer and growing concept in North America. For example, visiting companion dogs are being integrated into hospital environments to promote patient health. A recent controlled trial found a clinically significant decrease in patient pain following a 10-min visit with a dog in a hospital emergency department [78]. The trajectory toward recognition of the positive impact of companion animals in human health has likely been influenced in-part by the COVID-19 pandemic as well as Western social media (e.g., companion animals in human product advertisements). Companion animal-related stigma remains a concern though, especially in agriculture-based states and provinces. The lingering effects of the COVID-19 pandemic may offer a window of opportunity in Bangladesh to consider animal welfare as a result of attention being paid to their benefit in human lives during this particular time of need. This can be coupled with promotion of Bangladesh’s newly released 2019 *Animal Welfare Act*. A potential starting point is stray animal societies amenable to sharing frontline stories about the impacts of zooeyia. A key collaborator to also consider is the Livestock Service Department in Bangladesh because it requires assistance in promoting its work that is dependent on the *Animal Welfare Act*.
2. A specific human health area that highlights the presence of zooeyia is interpersonal violence. We know from the literature in North American that the important relationships individuals form with their companion animals can prevent them from leaving unsafe living conditions. A recent Canadian study of domestic violence shelter workers found that 77% of individuals did not leave abusive situations because they did not want to leave their companion animals behind [79]. It is well-established that there is “a link” between the abuse of humans and the abuse of animals [80]. Family and community violence is a concern in Bangladesh [81]. Recent statistics indicate that Bangladesh ranks 4th in the world in terms of violence against women by intimate partners [82]. Other research shows that over 70% of women face domestic violence in their lifetime [83]. Research also suggests that only 4% of victims report their abuse to police [84]. To our knowledge, there are no published studies in Bangladesh about violence against women and the link with companion animals. There is, however, literature suggesting that religious belief can influence behaviour and that companion animal ownership is lower among church attendees [85]. Paying attention to “the link” may be a novel way of increasing attention in the Bangladesh community about interpersonal and domestic violence and human health, and consequently influence discussion about animal welfare.
3. One Health is increasingly recognized today because of the COVID-19 pandemic, with near exclusive attention to zoonotic disease transmission, including in Bangladesh. For example, resent research has examined hygiene practices in Bangladesh refugee camps during the pandemic [86]. As shared, zooeyia is an important but under-recognized component of One Health. More specifically, the related One Welfare framework specifically considers animal welfare. According to Animal Health Canada, “One Welfare builds on the One Health concept and is a way to recognize the many social interconnections between human welfare, animal welfare and the integrity of the environment” [87]. Attention to One Welfare in Bangladesh alongside pandemic-related One Health work and scholarship may be an important means to consider animal welfare. One suggestion is to explore the influence of social media as there seems to be an existing platform to build off of.
4. There is much to be learned from across the globe about the potential to improve animal welfare by promoting practices that recognize zooeyia. For example, street dogs in Kampala, Uganda, are often killed to control diseases and their population [88] and the history of dogs is similar to that of Bangladesh—for hunting and security [89]. Meanwhile, in Northern Uganda, a project has trained street dogs to assist “survivors of war [to] cope with their trauma”, with the beneficial impacts of the human–animal bond highlighted [89]. In Canada, as another example, there is a human rights designation of emotional support animals across many provinces and territories. These are animals that support the mental health of their owners via the human–animal bond and these animals are allowed in rentals that are not designated to allow companion animals [90]. The human–animal bond is defined by the American Veterinary Association as “a mutually beneficial and dynamic relationship between people and animals that is influenced by behaviours essential to the health and wellbeing of both. This includes, among other things, emotional, psychological, and physical interactions of people, animals, and the environment” [90]. It recognizes the reciprocal nature of the human–companion animal relationship and thereby supports and extends the concept of zooeyia.

Despite the novelty and importance of the insights gathered in this exploratory study and their potential to inform companion animal welfare in Bangladesh, we recommend considering them with caution. Foremost, the small sample size of this study may not be representative of the entire companion-animal owning population in Bangladesh, or even the two targeted urban centres. However, this study is one of the first efforts of its type to identify the contemporary beneficial role of companion animals in human health and its potential to promote companion animal welfare in Bangladesh.

## Figures and Tables

**Table 1:** Socio-economic characteristics of companion-animal-owner study participants.

Characteristics	No. of Participants (%)
**Age (years)**	
18–30	10 (50)
31–50	8 (40)
50 and above	2 (10)
**Gender**	
Male	11 (55)
Female	9 (45)
**Marital status**	
Never married	9 (45)
Married	9 (45)
Divorced	1 (5)
Widower	1 (5)
**Education**	
Grades 10–12	5 (25)
Bachelors and above	15 (75)
**Religion**	
Muslim	15 (75)
Hindu	4 (20)
Christian	1 (5)
**Occupation**	
Employed	11 (55)
Unemployed	3 (15)
Homemaker	2 (10)
Student	4 (20)
**Duration of owning companion animals (years)**	
1–3	12 (60)
4–6	3 (15)
6 and above	5 (25)
**Household ownership status**	
Own house	7 (35)
Rented house	13 (65)
**Average monthly family income (USD)**	1369.81 (range: 171.41–10,713.49)

**Table 2:** Socio-demographic information of Animal Sellers and LSD officers.

Characteristics	Animal Sellers (N)	LSD Officers (N)
**Age (mean in years)**	31.70	47
**Sex**		
Male	10	5
Female	0	0
**Religion**		
Islam	7	4
Hinduism	2	1
Christianism	1	0
**Education**		
Below Grade 10	1	0
Grades 10–12	7	0
Bachelors and above	2	5
**Average duration at the current occupation (in years)**	8.4	21

## Data Availability

The data presented in this study are available on request from the corresponding author and the senior author. The data are not publicly available in deference to participants’ privacy in accordance with the approval of the Ethics Committee.

## Supplementary Materials

The following supporting information can be downloaded at: https://www.mdpi.com/article/10.3390/ani13091523/s1.
