# Supplementary material for: Recognizing Zooeyia to Promote Companion Animal Welfare in Urban Bangladesh
Source: Animals (Basel). 2023 May 1;13(9):1523. doi: 10.3390/ani13091523 (PMC10177534; doi:10.3390/ani13091523)
Supplement: Supplementary file 1 [file animals-13-01523-s001.zip › animals-2350403-supplementary.pdf]

INTERVIEW GUIDELINES সাক্ষাৎকার নির্দেশিকা

Examining the Social Benefits of Human-Animal Interaction in Urban Bangladesh
বাংলাদেশের শহর অঞ্চলে মানুষ এবং সঙ্গী প্রাণী-এর মিথষ্ক্রিয়ার সামাজিক সুফল অনুসন্ধান

Key Informant Interview Guideline for Household Members with Companion Animal যে সকল গৃহস্থলি সদস্যদের সঙ্গী প্রাণী আছে তাদের কেআইআই সাক্ষাৎকার নির্দেশিকা

1. Socio-demographic information জনমিতিক তথ্য

- ***a.*** Age, sex, marital status, educational status, occupational status, monthly family income, type of family (e.g. nuclear, joint, or extended family). বয়স, লিঙ্গ, বৈবাহিক অবস্থা, শিক্ষাগত যোগ্যতা, পেশা, মাসিক পারিবারিক উপার্জন, পরিবারের ধরণ (একক, যৌথ অথবা বর্ধিত পরিবার)
- ***b.*** Do you have any pets? আপনার কি কোন পোশা প্রাণী আছে?
- ***c.*** What is your pet (s) (e.g. dog, cat, rabbit, bird, and so on)? আপনার পোষা প্রাণীটি ধরণ কি (কুকুর, বিড়াল, খরগোশ, পাখি বা অন্যান্য)?
- ***d.*** How long have you been rearing your pet (s)? কত দিন যাবত আপনি এই প্রাণীটি পালন করছেন?

2. **History of Pet in the Society**

- a. Can you tell me about your history of having pet(s)? {Please probe-what pets have you lived with (e.g., cat, bird, dog, rabbit)? For how long? সমাজে পোষা প্রাণীর ঐতিহাসিক অবস্থান আপনার পোষাপ্রাণী পালনের প্রেক্ষাপট অনুগ্রহ করে আমাদেরকে বলবেন কি? (দয়া করে প্রোব করুন: কোন কোন প্রাণী পালন করেছেন, কতদনি যাবত পালন করেছেন?)
- b. What was the role of pets in your society in the past? (please probe details) আপনার সমাজে অতীতে পোষাপ্রাণীর ভূমিকা কি ছিল? (অনুগ্রহ করে বিস্তারিত জানুন)
- c. Is there any changes in the role of pet animals in your society compared to previous decades? If yes, please discuss the changes in detail. অতীতের সাথে তুলনা করলে আপনার সমাজে পোষাপ্রাণীর ভূমিকায় কোন ধরনের পরিবর্তন এসেছে কি? হ্যা হলে, পরিবর্তনের ধরণগুলি দয়া করে আলোচনা করুন?

3. Perceived Social Benefits of Having Pet(s) পোষা প্রাণী থাকার সামাজিক সুফল

- a. What is the roles of pet animals in your society? আপনার সমাজের বর্তমানে পোষাপ্রাণীর ভূমিকা কি?

b. What is the role of pets in your life? (please probe details about mental support, facilitating social interaction, stress management, etc.) অনুগ্রহ করে আপনার ব্যক্তিগত জীবনে পোষাপ্রাণীর ভূমিকা বণনা করুন। (প্রোব করুন: মানসিক সাপোর্ট, সামাজিক মিথস্ক্রিয়া, স্ট্রেস ম্যানেজমেন্ট, ইত্যাদি)

<u>Perceived Social Challenges of Having Pet (s) - Impact of HAI on Social Interaction Social Relationships পোষাপ্রাণী থাকার ফলে সামাজিক ঝুঁকি, সামাজিক মিথস্ক্রিয়া এবং সামাজিক সম্পর্কের উপর মানুষ- পোষা প্রাণী সম্পর্কের প্রভাব</u>

a. Who takes care of your pet? (please ask if there is anyone else in the household who takes care of the pet) আপনার পোষা প্রাণীটির রক্ষণাবেক্ষণের কাজ মূলত কে করেন? (আপনি ব্যতিত আর কেউ আছে কিনা যিনি এই প্রাণীটির/গুলির রক্ষণাবেক্ষণ করেন?)

b. What sort of care do you provide your pet? আপনার পোষা প্রাণীর কি কি ধরনের রক্ষণাবেক্ষনের কাজ আপনি করেন?

c. How much time do you spend daily with your pet? What do you do together? দৈনিক কতটা সময় আপনি আপনার পোষা প্রাণীর সঙ্গে কাটান? কি কি ভাবে প্রাণিটির সাথে আপনার সময় কাটে?

d. What was your initial goal (s) for having the pet (s)? (please probe the reasons in detail) পোষাপ্রাণী পালনের ক্ষেত্রে আপনার প্রাথমিক কি উদ্দেশ্য ছিল? (দয়া করে বিস্তারিত জানুন)

e. Do you know anyone who has pet (s)? If yes, how do you know him/them?আপনার পরিচিত আর কারো পোষা প্রাণী আছে কিনা আপনি জানেন কি? হ্যা হলে, আপনি কি ভাবে তার/তাদের সম্পর্কে জেনেছেন?

f. How often do you meet with person (s) with pet(s) together with your pets? আপনার পরিচিত যাদের পোষা প্রাণী আছে (যদি পরিচিত কেউ থেকে থাকে) তাদের সাথে আপনার পোষা প্রাণী সহ কত ঘন ঘন দেখা হয়?

g. Is there any organization/group in your area where people with pet (s) meet? আপনার এলাকায় এমন কোন সংগঠন/গ্রুপ আছে কি যেখানে পোষা প্রাণী সহ লোকজন একত্রিত হয়?

h. If yes, what are the activities you do there in the group? হ্য হলে, সেখানে দলগতভাবে কোন ধরনের কর্মকাণ্ড করেন কি?

i. Do you think your pet (s) is/are facilitating your interaction with other people? If yes, please discuss how? আপনি কি মনে করেন যে আপনার পোষা প্রাণী কোনভাবে সমাজের অন্য কোন মানুষের সাথে আপনার মিথস্ক্রিয়ায় প্রভাব ফেলে? হ্যা হলে, দয়া করে বিস্তারিত বলুন।

j. Do you face any social challenges for having your pet (s)? If yes, please discuss the challenge(s) you face (please probe- perception of people about pets in Bangladesh)? How do you mitigate the challenge(s)? গৃহপালিত প্রাণী থাকার কারণে আপনাকে কোন ধরনের সামাজিক অসুবিধার মধ্যে পড়তে হয় কি? হ্য হালে, দয়া করে বিস্তারিত বলুন- কি কি ধরনের অসুবিধা মোকাবিলা করতে হয়? (প্রোব করুন-পোষঅ প্রাণী সম্পর্কে বাংলাদেশের মানুষের ধারণা কি)? কিভাবে এই সমস্যাগুলি আপনি মোকাবিলা করেন?

k. What do you do if your pet(s) become sick? আপনার প্রাণীটি অসুস্থ হলে আপনি কি কি করেন?

Thank you very much for your time and participation. আপনার সময় এবং অংশগ্রহণের জন্য ধন্যবাদ

In-depth Interviews (IDI) with Pet Animal Shop Owners পোষা প্রাণী বিক্রেতাদের সাথে একান্ত সাক্ষাৎকার

1. Socio-demographic information - age, sex, educational status. জনমিতিক তথ্য- বয়স, লিঙ্গ, শিক্ষা
2. Tell us about your business. অনুগ্রহ করে আপনার পেশা সম্পর্কে আমাদেরকে বলুন
3. How long have you been involved in this business? এই পেশায় সাথে আপনি কতদিন ধরে জড়িত আছেন?
4. What sort of animals do you sell in your shop? আপনার এই দোকানে কি কি ধরনের প্রাণী বিক্রয় করেন?
5. Please explain the supply chain of these animals? এই সকল প্রাণীর উৎস সম্পর্কে দয়া করে আমাদেরকে বলুন
6. Do you maintain any record of sales of animals at your shop? If yes, please tell me আপনার দোকানে যে সকল প্রাণী বিক্রয় হয় তার কোন রেকর্ড কি রাখা হয়? হ্যা হলে, দয়া করে বলুন-

    a. How many pets do you sale on an average everyday? গড়ে দৈনিক কতগুলি পোশ প্রাণী আপনার দোকানে বিক্রয় হয়?
    b. Which pet (type of pet) is most popular in your locality? Why? কোন পোষা প্রাণীর চাহিদা আপনার দোকানে সবচেয়ে বেশি? কেন?
    c. How do you keep records (e.g. log books, database, etc.) of the sales of animals at your shop? আপনার দোকানে প্রাণী বিক্রয়ের তথ্য কিভাবে সংরক্ষণ করেন (লগ বুক, ডাটাবেস, ইত্যাদি)?

<u>Historical roles of companion animals (সঙ্গী প্রাণীর ঐতিহাসিক ভূমিকা)</u>

7. What do you think are the roles of pet animals in Bangladeshi society? বাংলাদেশের শহুরে সমাজব্যবস্থায় পোষা প্রাণীর ঐতিহাসিক ভূমিকাগুলি দয়া করে বিস্তারিত বলুন
8. What do you think are the general perceptions of people about having pets? পোষা প্রাণী সম্পর্কে আপনার সমাজের সাধারণ মানুষের ধারণা কি?
9. Is there any change(s) of trends in rearing pets in your country compared to the past? If yes, please explain the changes? অতীতের সঙ্গে তুলনা করলে আপনার সমাজে পোষা প্রাণী পালনের ক্ষেত্রে কোন পরিবর্তন এসেছে? হ্যা হলে, পরিবর্তনগুলি দয়া করে বর্ণনা করুন
10. Who are the most common customers of pet animals in your shop? [please probe: age(tentative), sex of the customers] আপনার দোকানে কোন ধরনের ক্রেতা সাধারণত পোষা প্রাণী ক্রয়ের জন্য আসেন? প্রোব করুন: ক্রেতাদের (আনুমানি)ক বয়স সীমা, লিঙ্গ]
11. What do you think are the most common reasons for rearing pet animals in your society?*(*probe details about the potential role of pets in healing mental illnesses, and creating

social networks) আপনার সমাজে পোষা প্রাণী পালনের প্রধান কারণগুলি কি কি? প্রোব করুন: মানসিক স্বাস্থ্য-বিষাদ গ্রস্থতা দূরীকরণে, সামাজিক যোগাযোগের বলয় সৃষ্টি, ইত্যাদি ক্ষেত্রে পোষা প্রাণীর কোন ভূমিকা আছে কিনা

12. What do you think are the common challenges of having pet in your society? আপনার সমাজে পোষা প্রাণী পালনের ক্ষেত্রে সাধারণত কি কি ধরনের অসুবিধা মোকাবিলা করতে হয়

Thank you very much for your time and participation. আপনার সময় এবং অংশগ্রহণের জন্য ধন্যবাদ

In-depth Interviews (IDI) with Livestock Service Officer লাইভস্টক সার্ভিস কর্মকর্তার একান্ত সাক্ষাৎকার

13. Socio-demographic information - age, sex, educational status, designation, length of service at this institution. জনমিতিক তথ্য- বয়স, লিঙ্গ, শিক্ষা, পদবি, চাকুরীর সময়কাল অনুগ্রহ করে
14. Please explain the supply chain of pet animals? পোষা প্রাণীর উৎস সমূহ সম্পর্কে বলুন
15. Is there any provision by the government to count the number of pets and pet suppliers? আপনার এলাকায় সরকারী উদ্যোগে পোষা প্রাণী পালনকারী এবং সরবরাহকারীদের কোন তালিকা প্রস্তুতির ব্যবস্থা আছে কি?

    a. If yes, please tell me details of the process? হ্যা হলে, অনুগ্রহ করে আমাদেরকে বিস্তারিত বলুন
    b. Which body of the government is responsible to conduct the census and keep the record? সরকারী কোন সংস্থা এই তালিকা প্রস্তুতির কাজ গুলি করে থাকে?
    c. What is the procedure to open a pet animal store in Bangladesh? বাংলাদেশে পোষা প্রাণী বিক্রয়ের দোকান করার জন্য কোন প্রক্রিয়া অনুসারণ করতে হয়?

16. What do you think are the roles of pet animals in Bangladeshi society? বাংলাদেশের শহুরে সমাজে পোষা প্রাণীর ভূমিকা কি বলে আপনি মনে করেন?
17. What do you think are the general perceptions of people about having pets? পোষা প্রাণী পালন সম্পর্কে সাধারণ মানুষের ধারণা কি বলে আপনি মনে করেন?
18. Is there any change(s) of trends in rearing pets in your country compared to the past? If yes, please explain the changes? অতীতের সঙ্গে তুলনা করলে আপনার সমাজে পোষা প্রাণী পালনের ক্ষেত্রে কোন পরিবর্তন এসেছে? হ্যা হলে, পরিবর্তনগুলি দয়া করে বর্ণনা করুন
19. Does your department keep any record of crime statistics involving animals? If yes, please tell me details about the process (please seek permission to see and take a copy of the records)? আপনার দপ্তরে প্রাণীর মাধ্যমে সন্ত্রাস নিয়ন্ত্রণ সংক্রান্ত কোন তালিকা কি সংরক্ষন করা হয়? হ্যা হলে দয়া করে

বিস্তারিত বলুন। (প্রোব করুন: তথ্যদাতার অনুমতি সাপেক্ষে ছবি তুলুন/ ফটোকপি করুন/ ই-কপি থাকলে সেটি/সেগুলি সংগ্রহ করুন)

20. Which people mostly rear pet(s) in your society? [please probe: age(tentative), sex of the customers] সমাজের কোন ধরনের মানুষ সাধারণত পোষা প্রাণী ক্রয়ের পালন করেন? প্রোব করুন: পালনকারীদের (আনুমানিক) বয়স সীমা, লিঙ্গ]

21. In your opinion, what are the most common reasons to have pets in your society? (Please probe- to get mental support from pets, creating social networks, commercial interest, etc.) আপনার মতে কি কি কারণে এই সমাজের মানুষ পোষাপ্রাণী পালন করেন? প্রোব: মানসিক সাপোর্ট, সামাজিক যোগাযোগের বলয় সৃষ্টি, অর্থনৈতিক স্বার্থে)

22. What do you think are the common social challenges to have pet at your home? পোষা প্রাণী পালনের কি কি ধরনের সামাজিক অসুবিধা আপনার এই সমাজে আছে বলে আপনি মনে করেন?

Thank you very much for your time and participation.
আপনার সময় এবং অংশগ্রহণের জন্য ধন্যবাদ।
